# Reproducibility of MRI-based white matter tract estimation using multi-fiber probabilistic tractography: effect of user-defined parameters and regions

**DOI:** 10.1007/s10334-021-00965-6

**Published:** 2021-10-18

**Authors:** Irène Brumer, Enrico De Vita, Jonathan Ashmore, Jozef Jarosz, Marco Borri

**Affiliations:** 1 Department of Neuroradiology, King’s College Hospital NHS Foundation Trust, Ground Floor, Ruskin Wing, Denmark Hill, London, SE5 9RS UK; 2 Department of Biomedical Engineering, School of Biomedical Engineering and Imaging Sciences, King’s College London, St Thomas’ Hospital, London, SE1 7EH UK; 3 Department of Medical Physics and Bioengineering, NHS Highland, Raigmore Hospital, Inverness, IV2 3UJ UK

**Keywords:** Diffusion magnetic resonance imaging, Reproducibility of results, Magnetic resonance imaging, Brain neoplasms

## Abstract

**Objective:**

There is a pressing need to assess user-dependent reproducibility of multi-fibre probabilistic tractography in order to encourage clinical implementation of these advanced and relevant approaches. The goal of this study was to evaluate both intrinsic and inter-user reproducibility of corticospinal tract estimation.

**Materials and methods:**

Six clinical datasets including motor functional and diffusion MRI were used. Three users performed an independent tractography analysis following identical instructions. Dice indices were calculated to quantify the reproducibility of seed region, fMRI-based end region, and streamline maps.

**Results:**

The inter-user reproducibility ranged 41–93%, 29–94%, and 50–92%, for seed regions, end regions, and streamline maps, respectively. Differences in streamline maps correlated with differences in seed and end regions. Good inter-user agreement in seed and end regions, yielded inter-user reproducibility close to the intrinsic reproducibility (92–97%) and in most cases higher than 80%.

**Discussion:**

Uncertainties related to user-dependent decisions and the probabilistic nature of the analysis should be considered when interpreting probabilistic tractography data. The standardization of the methods used to define seed and end regions is a necessary step to improve the accuracy and robustness of multi-fiber probabilistic tractography in a clinical setting. Clinical users should choose a feasible compromise between reproducibility and analysis duration.

## Introduction

The use of magnetic resonance imaging (MRI) for preoperative assessment and guidance during surgical procedures is becoming increasingly widespread in modern clinical settings. In the context of surgical interventions close to eloquent areas of the brain, functional magnetic resonance imaging (fMRI) combined with tractography are particularly valuable [1]. fMRI detects the areas of the brain activated during specific tasks, while tractography exploits the effect of tissue microstructure on the diffusion of water molecules to depict white matter fibers [2]. Tract estimation can be performed using different types of mathematical models, and with deterministic or probabilistic approaches [3, 4]. The classic diffusion tensor model [5] considers a unique diffusion direction for each voxel, and therefore makes the crude approximation that all fibers within a voxel are oriented in the same direction. Multi-fiber approaches can account for the presence of multiple fiber orientations within each voxel and thus yield a more accurate estimate of white matter tracts, especially in regions of complex fiber architecture [6–8]. Furthermore, probabilistic algorithms are able to account for the inherent uncertainty in the estimate of fiber orientation and provide superior sensitivity for reconstructing fiber bundles [9–11]. Neurosurgeons would benefit from more sensitive reconstructions to conservatively estimate the tract extent and proximity to pathology, as well as the degree of tract infiltration and involvement. This would serve as additional information to locate areas of risk that need attention or additional probing during surgery.

Commonly adopted presurgical planning systems almost exclusively implement diffusion tensor based deterministic tractography [9, 12] arguably because this is a more straightforward approach to the analysis, particularly for a clinical user.

Advanced preoperative evaluations with more sophisticated tractographic reconstructions are resource intensive and require collaboration between different clinical roles and expertise. At our neuroimaging centre, the complex image processing is performed by a medical physicist, and can take up to a few hours per case. The results are then reviewed with a radiologist, who reports the images, and the preoperative findings are finally discussed with the neurosurgeon before planning the surgery (and ideally are reviewed postoperatively). These logistics put time constraints on the process, with often a timescale of less than a week between image acquisition and surgery.

The robustness of the tractographic evaluation is a vital aspect in this process. Currently, there is a lack of published data on the user-dependent reproducibility of clinically applied probabilistic tractography, and a pressing need to assess this aspect in order to encourage clinical implementation of these advanced and relevant techniques. Probabilistic approaches both contain intrinsic variability at each run, due to the statistical nature of the analysis and the results, and multiple user-dependent decisions which can influence the final streamline distribution. The tractography seed and inclusion regions can be manually defined using anatomical landmarks, and in some cases can be informed by fMRI data [13, 14]. The use of fMRI data for the definition of seed and inclusion regions has been shown to increase the accuracy of tractography analysis [15, 16] and to allow separation of different tract components such as the hand and foot fibers of the corticospinal tract [1]. However, the variability of fMRI data is an additional factor to consider for the reproducibility of the tractography analysis.

In this work we evaluate both the intrinsic (run/re-run) and the inter-user reproducibility of corticospinal tract (CST) estimation using multi-fiber probabilistic tractography. We consider several factors influencing the reproducibility: the number of streamlines, the streamline density threshold used to determine the final streamline map, and the definition of the seed region and fMRI-based end regions.

## Materials and methods

### Subjects and MRI sequence protocol

Retrospective analysis of patient examinations was carried out with the approval of the institutional Clinical Audit Committee. Six clinical datasets acquired before tumor (*N *= 3) or epilepsy (*N* = 3) surgery close to the motor cortex were employed. Images were acquired at 1.5 T on a Siemens Magnetom Aera scanner (Siemens AG, Erlangen, Germany) using a 20-channel head/neck receive coil. The MRI sequence protocol consisted of a 3D T1-weighted MPRAGE for anatomy (TE/TR = 3.02/2200 ms, voxel size = (1 mm)^3^, FA = 8°, parallel imaging acceleration GRAPPA = 2), a gradient echo EPI sequence for fMRI (TE/TR = 40/3000 ms, voxel size = 2.5 × 2.5 × 3 mm^3^), and a spin echo EPI sequence for diffusion tractography (TE/TR = 86/9500 ms, voxel size = (2.5 mm)^3^, 6 baseline images at b = 0 s/mm^2^ and 64 diffusion directions at b = 1500 s/mm^2^). fMRI data were acquired for 6 cycles of alternating rest and activation periods of 30 s each, during the following motor tasks: finger tapping, foot rocking and lip pouting.

### Data analysis

The data were first visually checked for motion and Gibbs ringing artefacts, which were found to be limited. The analysis workflow made use of publicly available, advanced software packages and included recommended options and standards. fMRI data were processed using an in-house developed batch processing pipeline based on SPM12 (Wellcome Trust Centre for Neuroimaging, University College London, UK). Image pre-processing of fMRI data consisted of small motion correction (rigid body spatial transformation and least square algorithm, SPM12), non-linear co-registration with the anatomical volume (mutual information, SPM12) and isotropic Gaussian kernel smoothing (8 mm full width at half maximum). Diffusion data were reconstructed using constrained spherical deconvolution [17] (CSD) and probabilistic tractography in MRtrix3 [18] (version 0.3.14, http://www.mrtrix.org/). A single b-value (single-shell) response function of single-fiber white matter was computed [19] and a second-order integration over fiber orientation distributions (iFOD2) streamline generation algorithm was employed [20].

**Fig. 1:**
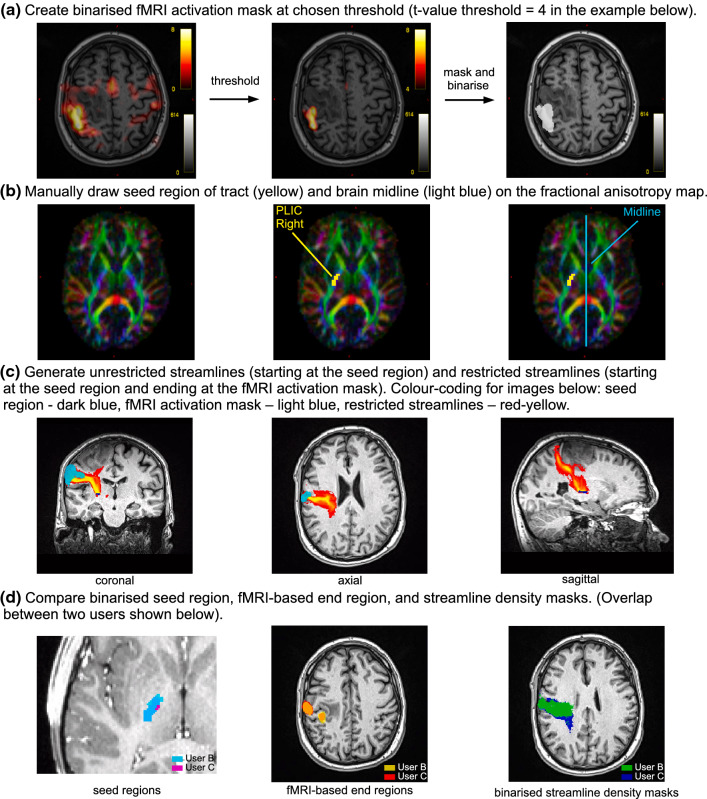
Workflow for generation of streamlines using fMRI and diffusion MRI data (**a**–**c**) and subsequent inter-user comparison (**d**)

Three users (medical physicists) with different experience in MR image processing and tractography analysis (user A: in training, less than a year for both; user B: 9 and 8 years, respectively; user C: 10 and 2 years, respectively) performed a blind and independent data analysis with the following instructions:

1. For each motor fMRI task, apply a threshold to the activation map, in order to spatially isolate the area corresponding to the highest activation in the relevant cortical area and convert it into an activation mask (Fig. 1a).
2. Combine the masks from all motor tasks to form a single mask encompassing the motor activation area.
3. On an axial slice of the fractional anisotropy map, manually draw the CST seed region on the posterior limb of the internal capsule (PLIC) in the hemisphere of interest, including only voxels with predominant diffusion in a superior-inferior direction (Fig. 1b). Additionally, define an exclusion region by positioning a sagittal plane through the inter-hemispheric fissure (midline), to avoid streamlines crossing over to the opposite hemisphere.
4. Generate streamlines using only the seed region (unrestricted streamlines) and using both the seed region and the fMRI-based activation mask as end region (restricted streamlines) [21] (Fig. 1c).

The algorithm generated streamlines from the seed region until 100,000 streamlines had been selected considering both inclusion and midline exclusion regions. User-dependent inputs which can affect the final streamline distribution included the definition of the seed and end regions. The spatial extent of the end regions is affected by both the choice of threshold and the selection of the activated area in the fMRI maps. Figure1d shows the region and streamline results obtained by two different users for the same patient.

### Reproducibility assessment

Streamline density maps (tractograms representing the number of streamlines per voxel) were generated using the streamlines produced by the probabilistic tractography analysis. To assess the reproducibility of probabilistic tractography at a set streamline density, the streamline density maps were thresholded and binarized by applying a density threshold [22]. Pairs of binarized maps (*α* and *β*) were then compared using the Dice index (DI) [23]:

1 $${\text{DI}}\; = \;\frac{{2 * \left( {\alpha \cap \beta } \right)}}{\alpha + \beta }$$

Initially, the streamline density threshold was set to 2 × 10^–4^ (i.e. 20 over 100,000 selected streamlines for a voxel size of 1mm^3^), matching the value used clinically at our institution.

**Fig. 2:**
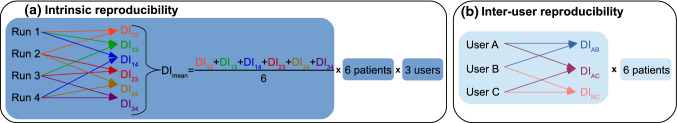
(**a**) For the intrinsic reproducibility, a mean Dice index was calculated for each patient-user combination by averaging over the Dice indices *DI*_*ij*_ obtained for all possible comparisons between the four runs performed (with *i*,* j* indices indicating the run number, and *DI*_*ij*_ being equivalent to *DI*_*ji*_). (**b**) For the inter-user reproducibility, the results obtained by different users were compared pair-wise, resulting in three Dice indices *D*_*kw*_ for each patient (with *k*,* w* indices indicating the ‘user’, and *DI*_*kw*_ being equivalent to *DI*_*wk*_)

For the intrinsic reproducibility resulting from the probabilistic nature of the tract estimation (run/re-run), four analysis runs with identical parameters and specified regions were performed for each patient-user combination (Fig. 2a). Results were then compared across the four runs, resulting in a total of six DIs, which were averaged to yield a mean DI for each patient-user combination.

To evaluate the inter-user reproducibility, the binarised streamline density masks obtained by the three different users were compared pair-wise for each patient (Fig. 2b). DI analysis was also applied to the seed region and fMRI-based activation masks, in order to evaluate the influence of these components separately. Pearson’s correlation coefficients (PCCs) were calculated to investigate the correlation between different sets of DIs. Multiple linear correlation was also performed using the seed and end regions’ DIs as predictors for the DIs of restricted streamlines in order to determine the combined effect of seed and end regions.

Finally, run/re-run reproducibility analysis was repeated for all patients and users varying both the streamline density thresholds (range 1–40 × 10^–4^) and the number of selected streamlines (range 10,000–250,000), to assess the dependence of the intrinsic reproducibility on these parameters as well as the duration of the analysis in different conditions. Intrinsic reproducibility DIs were also simulated using downsampled data generated by extracting subsets randomly choosing a selection of streamlines from a 1,000,000 streamline dataset in a representative case (patient 1, user A).

All correlations were calculated in MATLAB Version R2018a (The MathWorks Inc, Natick, Massachusetts) and the significance level was set to *p* < 0.05.

## Results

### Intrinsic reproducibility (run/re-run, 100,000 streamlines, 2 × 10^–4^ threshold)

**Fig. 3:**
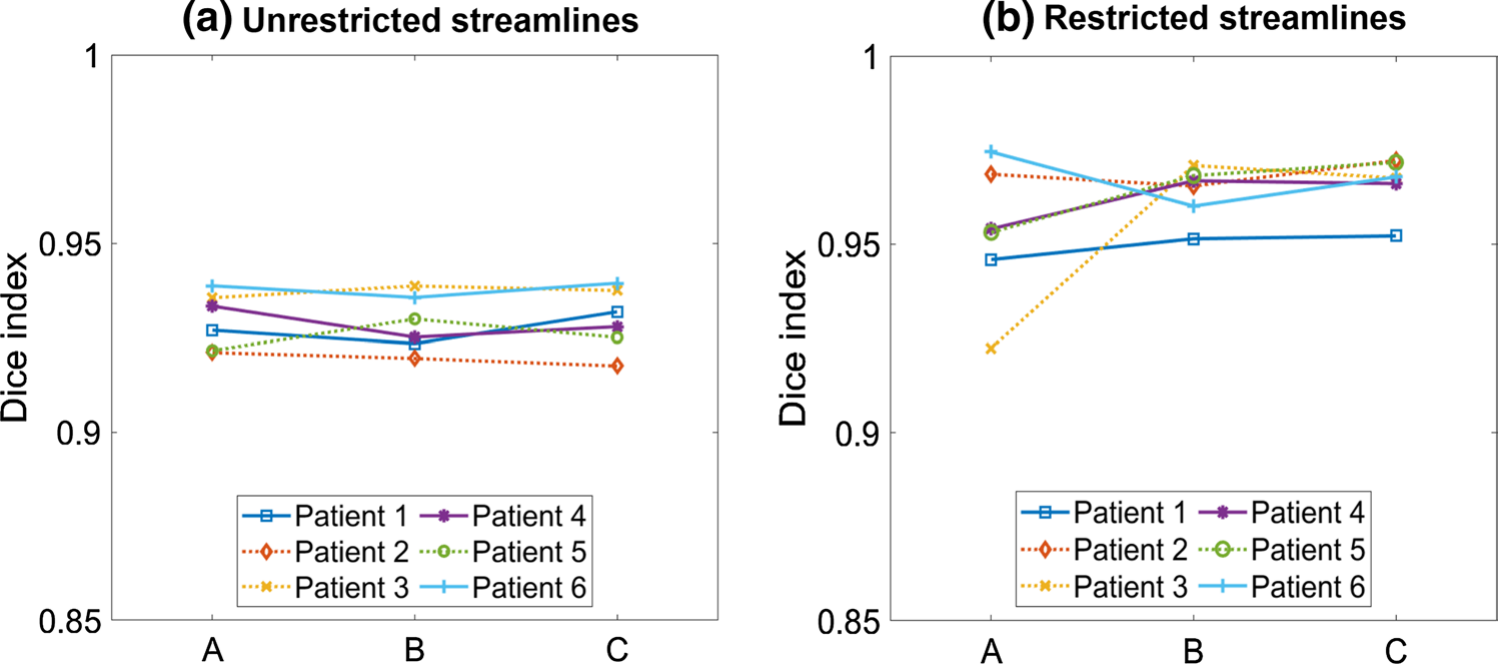
Intrinsic reproducibility of (**a**) unrestricted and (**b**) restricted streamline maps quantified by DIs averaged over four identical analysis runs for the three users for each of the six patients. The epilepsy and tumor patients are represented with dashed and solid lines, respectively

DIs obtained for each patient-user combination ranged 0.92–0.94 and 0.92–0.97 for the unrestricted and restricted streamline maps, respectively (Fig. 3). Intrinsic reproducibility was generally higher for restricted streamline maps than for unrestricted ones, probably due to the fact that additional constraints limit the number of possible pathways.

### Inter-user reproducibility (100,000 streamlines, 2 × 10^–4^ threshold)

**Fig. 4:**
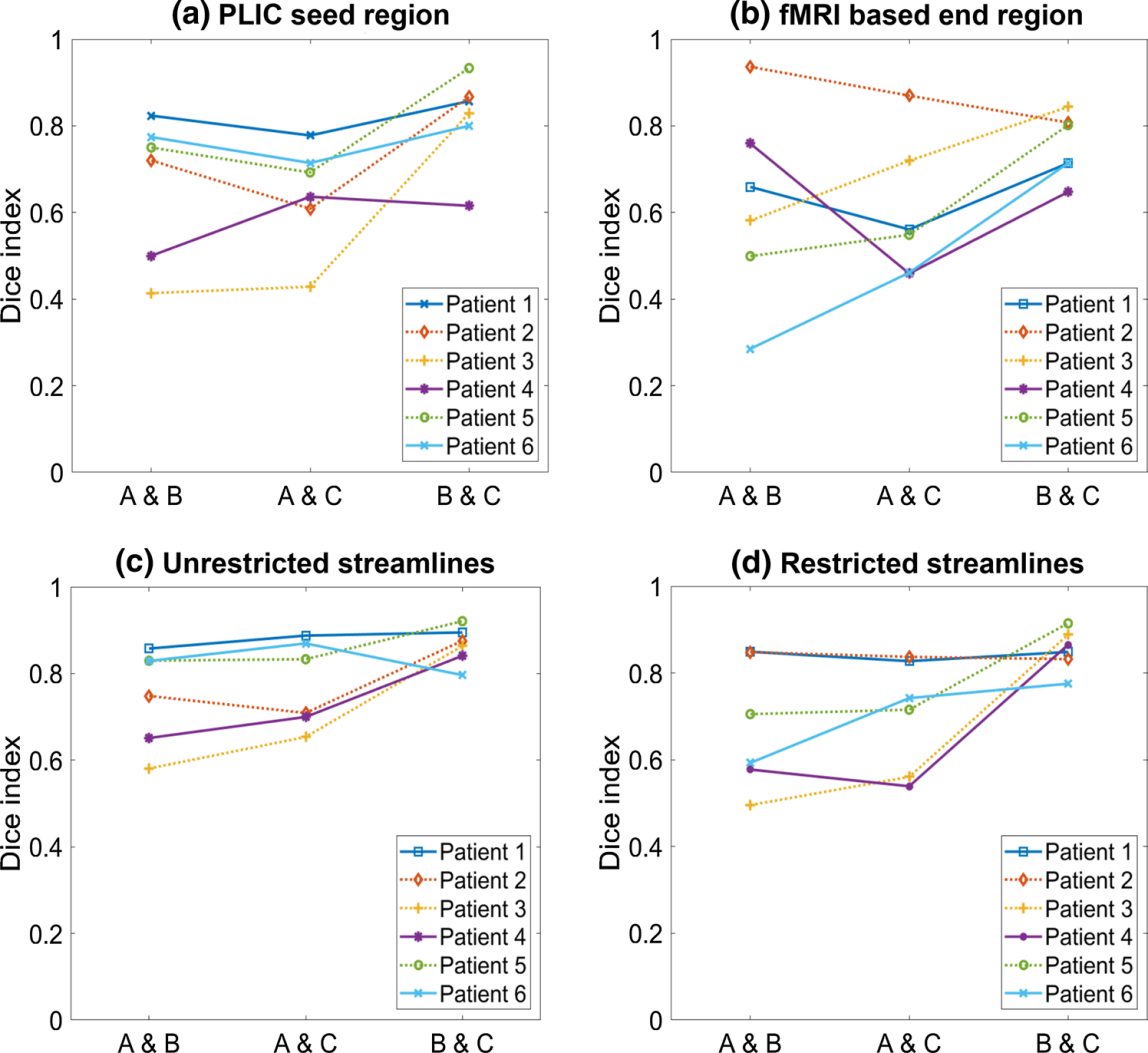
Inter-user reproducibility of (**a**) seed region, (**b**) end region, (**c**) unrestricted streamline maps and (**d**) restricted streamline maps quantified by Dice indices calculated for all user pairs and for each of the six patients. The epilepsy and tumor patients are represented with dashed and solid lines, respectively. Note the range of the y axis, which is different than in Fig. 3

DIs obtained for each user pair across all patients are shown in Fig. 4 for seed region, fMRI-based end region, unrestricted and restricted streamline maps. DIs ranged 0.41–0.93 for the seed region, 0.29–0.94 for the fMRI-based end region, 0.58–0.92 for the unrestricted streamline maps, and 0.50–0.92 for the restricted streamline maps. Figure 4 shows that the DIs had a spread of values across patients, and this was dependent on the user pair. Comparisons between users B and C have DIs with higher value and smaller variation, indicating greater overlap and consistency. Larger variations in seed and end region DIs (Fig. 4a and b) corresponded to larger variations in streamline DIs (Fig. 4c and d). This suggests that smaller differences in user-defined regions lead to improved reproducibility.

Unrestricted streamline maps showed a high correlation with seed regions (PCC = 0.90). For restricted streamline maps, there was a significant correlation for both seed and end regions individually (PCCs were 0.73 and 0.57, respectively) and the correlation found with the multiple linear regression model, which considered both seed and end regions, was also significant (PCC = 0.87). This verifies that the inter-user reproducibility of restricted streamline maps has a dependence on the definition of seed and end regions.

Regarding the seed regions, only the two-dimensional overlap between the regions defined by different users was considered. It was found that the seed regions were defined in different slices in four out of six patients, a factor which might contribute to reduced overlap. The correlation between DIs and the difference in seed region position (defined as the number of slices separating the two regions) was investigated, and a significant negative correlation for seed regions, unrestricted, and restricted streamline maps was found (PCCs were − 0.73, − 0.75, and − 0.72, respectively). This indicates that a larger separation between seed region slices yielded a lower inter-user reproducibility.

### Intrinsic reproducibility as a function of number of streamlines and density threshold

**Fig. 5:**
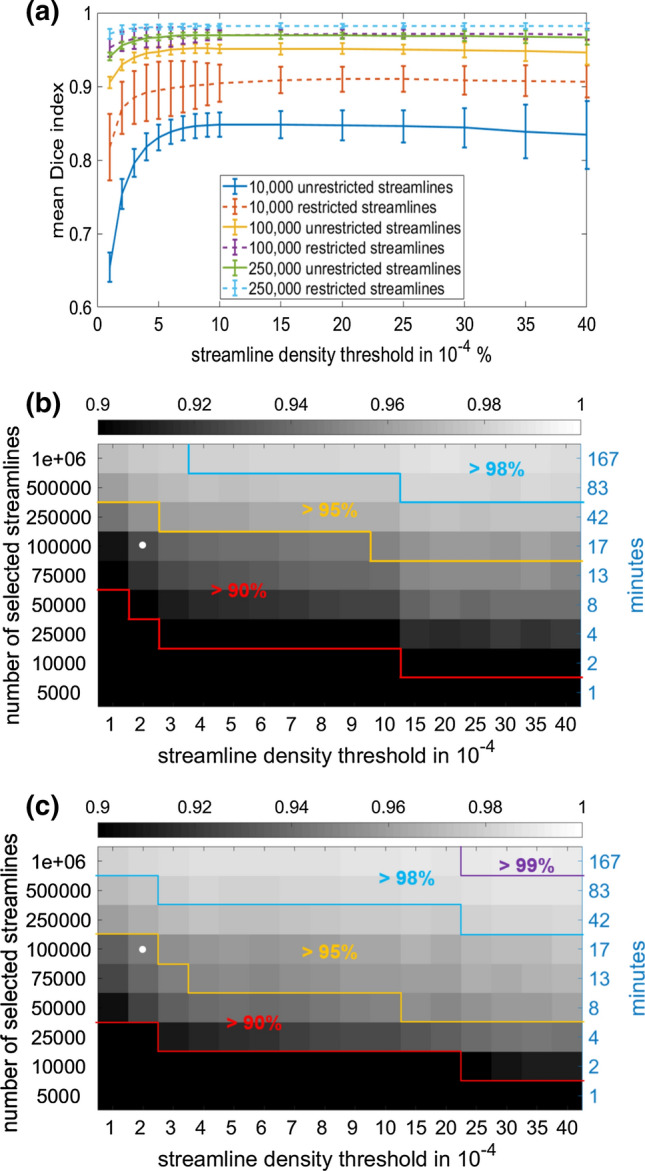
**(a)** Dependence of the Dice index on the streamline density threshold chosen to visualize the final tractogram for different number of selected streamlines. The values shown are the mean Dice indices obtained by averaging over all eighteen available patient-user combinations. The error bars indicate the standard deviation of the mean Dice index distributions. (**b**) Dependence of the intrinsic reproducibility of unrestricted streamline maps on the number of selected streamlines, the streamline density threshold and the duration of the analysis. (**c**) Dependence of the intrinsic reproducibility of restricted streamline maps on the number of selected streamlines, the streamline density threshold and the duration of the analysis. The white dot in (**b**) and (**c**) correspond to the case of 100,000 selected streamlines and a streamline density threshold of 2 × 10^–4^ (setting used for evaluating the inter-user reproducibility)

The DI curves in Fig. 5a were averaged over all patients and users and show the dependence of DIs on the streamline density threshold for different number of selected streamlines. In all curves, DIs increased for threshold values between 1 and 10 × 10^–4^, while at higher thresholds they either reached a plateau or showed a slow decrease for values higher than 30 × 10^–4^ (blue and yellow curves in Fig. 5a). In all conditions, restricted streamline maps yielded higher DIs, as also found in Fig. 3. For low thresholds and number of streamlines, DIs can reach suboptimal values (< 0.75). Increasing the number of streamlines increased analysis time and reproducibility, and decreased differences across users and patients (represented by the standard deviation). The relationship between number of streamlines, reproducibility, and analysis duration is shown in Fig. 5b and c using the simulated data. Figure 5b and c illustrate the fact that, for a chosen threshold, users need to find a clinically feasible compromise between reproducibility and analysis time.

## Discussion

Accurate and reproducible tract estimation obtained from advanced image processing such as tractography is crucial for presurgical planning. In this work we investigate the factors influencing the reproducibility of white matter tractograms generated using multi-fiber probabilistic tractography. In particular, we highlight that this analysis requires two considerations:

- 1) Different runs of the same analysis with identical seed and end regions will produce similar, but not identical, streamline density maps. The degree of similarity between the produced maps is affected by the chosen minimum number of streamlines selected.
- 2) The appearance of the streamline density map that a clinical user (e.g. neuroradiologist, neurosurgeon) sees, and particularly its spatial extent, depends on the lowest density displayed (density threshold). The reproducibility of the maps thus has a dependence on the streamline density threshold.

Although higher streamline densities indicate higher confidence in the presence of a fiber bundle and are more reproducible (Fig. 5), in a clinical setting the map provided to the neurosurgeon should have conservatively larger margins (i.e. it should contain voxels at lower density). In CST evaluations at our institution we set this threshold to 2 × 10^–4^. This provides a good compromise between removing false positive streamlines whilst still minimizing any false negative pathways [12] which can have serious clinical implications [13].

The intrinsic reproducibility of probabilistic tractography is purely due to the statistical nature of the analysis, and represents the upper limit of reproducibility. Although increasing the number of selected streamlines progressively reduces statistical variability [24], users should find a good compromise between the duration of the processing and the achievable reproducibility (Fig. 5). For the values adopted in this analysis for the CST (100,000 streamlines, 2 × 10^–4^ threshold), the intrinsic reproducibility is > 92%, but it can be as low as 75% for 10,000 streamlines. The analysis with 100,000 streamlines requires 20 min per case when including both restricted and unrestricted streamlines (iMac, Processor 3.3 GHz Intel Core i5, RAM 32 GB 1867 MHz DDR3), which is a sensible trade-off when results need to be both accurate and prompt. For interactive modifications of the analysis 10,000 streamlines (2 min, 75%-90% reproducibility) can facilitate a dynamic discussion whilst still maintaining acceptable streamline map reproducibility. Such discussions would not be possible for 1,000,000 streamlines (3 h, > 97% reproducibility) particularly since imaging is often undertaken just days prior to surgery.

Although increasing the number of streamlines (keeping all other conditions constant) also generally increases inter-user reproducibility, this is significantly affected by the definition of seed and end regions (Fig. 4), with values as low as 58% for unrestricted streamline maps and 50% for restricted ones. However, where users have good agreement (users B and C), the inter-user reproducibility is higher than 80% and close to intrinsic reproducibility. Users B and C are more experienced in tractography processing and this might have led to more coherent choices, highlighting the importance of standardizing the region definitions.

In our analysis, the comparison between unrestricted and restricted streamline maps shows the effects of further inclusion criteria on intrinsic and inter-user reproducibility. We have considered both unrestricted and restricted streamlines as they provide complementary information in clinical evaluations: while restricted streamlines are able to differentiate hand, foot, and lips CST branches, unrestricted streamlines are less specific but very sensitive for investigation of potential infiltration by the tumor. However, the unrestricted streamlines include all possible streamlines originating from the seed region, and, therefore, an expert user is needed to disregard potentially spurious streamlines, especially in clinical evaluations.

In this paper we show that several factors affect the final streamline map, however, uncertainty remains regarding which generated tractogram represent the ground truth as we did not compare results to a gold standard. Complete validation via subcortical mapping [9] or comparison to cytoarchitectonic maps [12] was outside the scope of this research.

Previous studies have looked into the inter-user reproducibility of tractography, noting that user performance is an important limiting factor. However, most studies assessed reproducibility in relation to apparent diffusion coefficient, fractional anisotropy, mean diffusivity, turning angle threshold, and streamline or voxel count [25–29]. More recently, a study looked at optimizing the number of selected streamlines through mathematical models aiming to reduce the reproducibility of probabilistic tractography but did not consider the influence of user-dependent choices [30]. Another recent work looked at whole brain streamline reproducibility between users by comparing binarized streamline volumes, but using deterministic and atlas-driven approaches [31]. Automated pipelines for generation of white matter fibers have been proposed [32, 33]. These approaches based on automatic segmentation do not require user input, and therefore do not suffer from intra- or inter-user variability. However, they mostly rely on image registration and pre-determined atlases, and are therefore strongly affected by abnormal anatomy (e.g. the presence of a large lesion) [34]. As a result, they are generally applied to cases where structural alteration due to pathology is absent or minimal [24]. Furthermore, such approaches require tools and resources that are normally not available in a clinical setting. Another recent study assessed the reproducibility of bundle segmentation and found a large variability across and within protocols, highlighting the lack of standardization in this type of analysis [10].

Our paper considers the practical choices that a clinical user makes to reconstruct the CST in a typical setting, and focuses on assessing how these choices influence the variability of the visual information presented for presurgical planning. Our results demonstrate that this variability can be quite substantial, but also that it can be minimized with appropriate choices.

Limitations of this study are the small number of clinical datasets for which the analysis from three users was available, and evaluation of the CST only. Nevertheless, this pilot dataset is sufficient to illustrate how streamline map reproducibility is affected by a range of parameters in a representative number of situations. Furthermore, as we have shown for the intrinsic reproducibility, it is possible to extend the analysis to any streamline density threshold or number of streamlines. However, for clarity, in this paper we have only reported the inter-user reproducibility values for the parameters we are currently using in our clinical setting [12]. No pre-processing steps (denoising, eddy current distortion correction, Gibbs ringing artefact reduction) were applied to the data presented here as not available in the software packages used at the time of analysis. Users are encouraged to apply these steps to improve the quality of the data. Finally, when correlating the Dice indices, we did not correct for multiple comparisons as it is not trivial in this setting. Although the single tractograms/regions are used more than once in the analysis, each Dice index quantifies the overlap between two of them, and is therefore unique.

fMRI-based end regions substantially contribute to the final streamline distribution, and their spatial extent is affected by arbitrary user choices, like the statistical activation threshold and the identification of the activated areas. Future work should aim at standardizing, or, ideally, automating these choices, as this would significantly improve the reproducibility of the restricted streamline maps. Furthermore, we plan to extend this work to larger cohorts and other tracts, as well as evaluate the impact of reproducibility on surgery-related decisions and use intraoperative findings for validation.

## Conclusions

In this work, we assessed the inter-user and intrinsic reproducibility of white matter tract estimation using multi-fiber probabilistic tractography. This work emphasizes that inter-user differences in seed and end regions should be minimized to improve the reproducibility of the estimation of unrestricted and restricted streamline maps. However, despite the influence of these factors, it was shown that in most cases streamline map reproducibility was higher than 60% and it was possible to reach optimal reproducibility (70–90%) between users for good agreement of seed and end regions. Furthermore, this paper, through representative examples, offers guidance towards reaching a feasible compromise between duration of analysis and achievable reproducibility in a clinical setting.

This study demonstrates that the uncertainties related to the user-dependent choices (threshold for fMRI activation mask and position of seed region), the streamline density threshold chosen to visualize the streamline maps, and the probabilistic nature of the analysis should be considered when interpreting probabilistic tractography data. The standardization of the methods to define the seed region (particularly the slice chosen) and the fMRI end regions is a necessary step to improve the robustness of the visual information provided by multi-fiber probabilistic tractography for presurgical planning in the clinical routine.

## References

[1] Smits M, Vernooij MW, Wielopolski PA, Vincent AJPE, Houston GC, van der Lugt A (2007). Incorporating functional MR imaging into diffusion tensor tractography in the preoperative assessment of the corticospinal tract in patients with brain tumors. AJNR.

[2] Bizzi A (2009). Presurgical mapping of verbal language in brain tumors with functional MR imaging and MR tractography. Neuroimaging Clin.

[3] O’Donnell LJ, Westin CF (2011). An introduction to diffusion tensor image analysis. Neurosurg Clin.

[4] Essayed WI, Zhang F, Unadkat P, Cosgrove GR, Golby AJ, O'Donnell LJ (2017). White matter tractography for neurosurgical planning: a topography-based review of the current state of the art. NeuroImage Clin.

[5] Basser PJ, Mattiello J, LeBihan D (1994). Estimation of the effective self-diffusion tensor from the NMR spin echo. JMR Ser B.

[6] Lazar M (2010). Mapping brain anatomical connectivity using white matter tractography. NMR Biomed.

[7] Descoteaux M, Deriche R, Knosche TR, Anwander A (2008). Deterministic and probabilistic tractography based on complex fibre orientation distributions. IEEE Trans Med Imaging.

[8] Farquharson S, Tournier JD, Calamante F, Fabinyi G, Schneider-Kolsky M, Jackson GD, Connelly A (2013). White matter fiber tractography: why we need to move beyond DTI. J Neurosurg.

[9] Bucci M, Mandelli ML, Berman JI, Amirbekian B, Nguyen C, Berger MS, Henry RG (2013). Quantifying diffusion MRI tractography of the corticospinal tract in brain tumors with deterministic and probabilistic methods. NeuroImage Clin.

[10] Schilling KG, Rheault F, Petit L, Hansen CB, Nath V, Yeh FC et al (2021) Tractography dissection variability: what happens when 42 groups dissect 14 white matter bundles on the same dataset? NeuroImage 243:118502. 10.1016/j.neuroimage.2021.118502 10.1016/j.neuroimage.2021.118502 PMC8855321 34433094

[11] Maier-Hein KH, Neher PF, Houde JC, Côté MA, Garyfallidis E, Zhong J (2017). The challenge of mapping the human connectome based on diffusion tractography. Nat Commun.

[12] Ashmore J, Pemberton HG, Crum WD, Jarosz J, Barker GJ (2020). Implementation of clinical tractography for pre-surgical planning of space occupying lesions: an investigation of common acquisition and post-processing methods compared to dissection studies. Plos One.

[13] Kinoshita M, Yamada K, Hashimoto N, Kato A, Izumoto S, Baba T, Maruno M, Nishimura T, Yoshimine T (2005). Fiber-tracking does not accurately estimate size of fiber bundle in pathological condition: initial neurosurgical experience using neuronavigation and subcortical white matter stimulation. Neuroimage.

[14] Dimou S, Battisti RA, Hermens DF, Lagopoulos J (2013). A systematic review of functional magnetic resonance imaging and diffusion tensor imaging modalities used in presurgical planning of brain tumour resection. Neurosurg Rev.

[15] Schonberg T, Pianka P, Hendler T, Pasternak O, Assaf Y (2006). Characterization of displaced white matter by brain tumors using combined DTI and fMRI. Neuroimage.

[16] Kleiser R, Staempfli P, Valavanis A, Boesiger P, Kollias S (2010). Impact of fMRI-guided advanced DTI fiber tracking techniques on their clinical applications in patients with brain tumors. Neuroradiology.

[17] Tournier J-D, Calamante F, Gadian DG, Connelly A (2004). Direct-estimation of the fiber orientation density function from diffusion-weighted MRI data using spherical deconvolution. Neuroimage.

[18] Tournier J-D, Smith R, Raffelt D, Tabbara R, Dhollander T, Pietsch M, Christiaens D, Jeurissen B, Yeh C-H, Connelly A (2019). MRtrix3: a fast, flexible and open software framework for medical image processing and visualisation. Neuroimage.

[19] Tournier J-D, Calamante F, Connelly A (2013). Determination of the appropriate b value and number of gradient directions for high-angular-resolution diffusion-weighted imaging. NMR Biomed.

[20] Tournier J-D, Calamante F, Connelly A (2010) Improved probabilistic streamlines tractography by 2nd order integration over fibre orientation distributions? In: Proceedings of the 18th scientific meeting, International Society for Magnetic Resonance in medicine, Stockholm pp 1670

[21] Conturo TE, Lori NF, Cull TS, Akbudak E, Snyder AZ, Shimony JS, McKinstry RC, Burton H, Raichle ME (1999). Tracking neuronal fiber pathways in the living human brain. Pro Natl Acad Sci.

[22] Brumer I, De Vita E, Ashmore J, Jarosz J, Borri M (2019) How reproducible are the results of probabilistic white matter tract estimation? In: Proceedings of the 27th scientific meeting, International Society for Magnetic Resonance in medicine, Montréal pp 3653

[23] Dice LR (1945). Measures of the amount of ecologic association between species. Ecology.

[24] Toosy AT, Ciccarelli O, Parker GJ, Wheeler-Kingshott CA, Miller DH, Thompson AJ (2004). Characterizing function–structure relationships in the human visual system with functional MRI and diffusion tensor imaging. Neuroimage.

[25] Wakana S, Caprihan A, Panzenboeck MM, Fallon JH, Perry M, Gollub RL, Hua K, Zhang J, Dubey P, Blitz A, van Zijl P, Mori S (2007). Reproducibility of quantitative tractography methods applied to cerebral white matter. Neuroimage.

[26] Ozturk A, Sasson AD, Farrell JAD, Landman BA, da Motta ACBS, Aralasmak A, Yousem DM (2008). Regional differences in diffusion tensor imaging measurements: assessment of intrarater and interrater variability. AJNR.

[27] Paldino MJ, Hedges K, Rodrigues KM, Barboriak DP (2014). Repeatability of quantitative metrics derived from MR diffusion tractography in paediatric patients with epilepsy. Br J Radiol.

[28] Brandstack N, Kurki T, Laalo J, Kauko T, Tenovuo O (2016). Reproducibility of tract-based and region-of-interest DTI analysis of long association tracts. Clin Neuroradiol.

[29] Rheault F, De Benedictis A, Daducci A, Maffai C, Tax CMW, Romascano D, Caverzasi E, Morency FC, Corivetti F, Pestilli F, Girard G, Theaud G, Zemmoura I, Hau J, Glavin K, Jordan KM, Pomiecko K, Chamberland M, Barakovic M, Goyette N, Poulin P, Chenot Q, Panesar SS, Petit L, Descoteaux M (2020). Tractostorm: the what, why, and how of tractography dissection reproducibility. Hum Brain Map.

[30] Reid LB, Cespedes MI, Pannek K (2020). How many streamlines are required for reliable probabilistic tractography? Solutions for microstructural measurements and neurosurgical planning. Neuroimage.

[31] Bayrak RG, Wang X, Schilling KG, Greer JM, Hansen CB, Blaber JA, Williams O, Beason-Held LL, Rogers BP, Landman BA (2020) TractEM: fast protocols for whole brain deterministic tractography-based white matter atlas. Cold Spring Harbor Laboratory. 10.1101/651935

[32] Mancini M, Vos SB, Vakharia VN, O'Keeffe TK, Barkhof F, Dorfer C, Soman S, Winston GP, Wu C, Duncan JS, Sparks R, Ourselin S (2019). Automated fiber tract reconstruction for surgery planning: extensive validation in language-related white matter tracts. NeuroImage Clin.

[33] O'Donnell LJ, Suter Y, Rigolo L, Kahali P, Zhang F, Norton I, Albi A, Olubiyi O, Meola A, Essayed WI, Unadkat P, Ciris PA, Wells WM, Rathi Y, Westin C-F, Golby AJ (2016). Automated white matter fiber tract identification in patients with brain tumors. Neuroimage Clin.

[34] Rheault F, Poulin P, Caron AV, St-Onge E, Descoteaux M (2020). Common misconceptions, hidden biases and modern challenges of dMRI tractography. J Neural Eng.

